# Squamous Differentiation and Cytokeratin Expression in an Osteosarcoma: A Case Report and Review of the Literature

**DOI:** 10.4137/cpath.s582

**Published:** 2008-03-18

**Authors:** Lester J. Layfield, Lyska Emerson, Julia R. Crim, Lor Randall

**Affiliations:** 1 Departments of Pathology; 2 Radiology and; 3 Orthopedic Surgery (Sarcoma Service) University of Utah Hospital and Clinics, Salt Lake City, Utah, 84132

**Keywords:** osteosarcoma, cytokeratin, squamous differentiation

## Abstract

Cytokeratin expression has been documented in a variety of sarcomas including synovial sarcomas, epithelioid sarcomas, Ewing’s sarcomas and, rarely, osteosarcomas. In osteosarcomas immunohistochemically shown to expression cytokeratins, a component of epithelioid cells is generally present. These epithelioid cytokeratin positive cells raise the possibility of metastatic disease with prognostic and therapeutic implications differing from primary osteosarcoma. The cytokeratin-expressing cells of the cases reported in the literature have not shown definitive squamous differentiation with keratin pearl formation. We report a case of osteosarcoma in which islands of malignant squamous cells were present showing keratin pearl formation and expression of cytokeratins.

## Introduction

Cytokeratin expression has been documented by immunohistochemistry in a variety of sarcomas^1^ including synovial sarcoma.^2^–^9^ Only synovial sarcomas and epithelioid sarcomas are believed to show true epithelial differentiation, while the other sarcomas express so-called “anomalous” cytokeratins.^1^ Cytokeratins have been rigorously classified by Moll et al.^10^ In sarcomas with “anomalous” cytokeratin expression, the cytokeratin is expressed by a minority of cells and is present in only a portion of the cytoplasm.^1^ Most of the sarcomas with “anomalous” cytokeratin expression only express cytokeratins 8 and 18 as detected by CAM 5.2. Cytokeratin expression has been documented in osteoblastic, fibroblastic and chondroblastic osteosarcomas.^2^ Cytokeratin is most strongly and extensively expressed in osteosarcomas with an extensive epithelioid cell component.^2^ Cytokeratin in these cases is detected by antibodies AE1,3 and is present only in the epithelioid cells. AE1,3 is an antibody mixture reactive against cytokeratins 56.5, 50, 51, 48, 40, 67, 66, 65, 64, 59, 58, 56 and 52 KD as described by Moll et al.^10^ These epithelial osteosarcomas contain a component of cells with eccentrically located vesicular nuclei, prominent nucleoli and abundant pale eosinophilic cytoplasm.^6^ Such osteosarcomas require distinction from metastases to bone. This is usually achieved by excluding an epithelial primary elsewhere and demonstration of malignant osteoid. The majority of cases reported do not produce clearly identifiable epithelial structures such as glands, ducts or squamous epithelium with dykeratotic cells or keratin pearl formation.^2^–^9^ Some osteosarcomas expressing cytokeratins have demonstrated rosette-like structures.^6^

We report a case of osteosarcoma in which a minority population of cells demonstrated striking differentiation towards squamous epithelium with dykeratotic cells and keratin pearls. The cells reacted with antibodies against AE1,3. This squamous differentiation made distinction from metastatic disease difficult. Herein we report the clinical and pathologic findings in this case.

## Case Report

**Figure 1.**
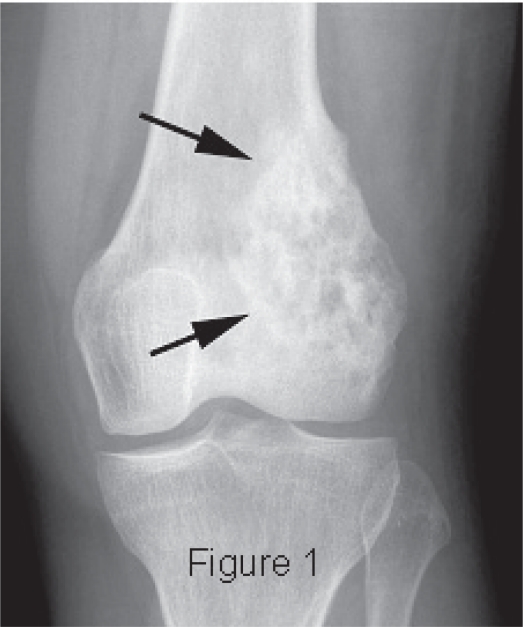
Radiograph showing an osteolytic and osteoblastic intra-medullary tumor characteristic of osteosarcoma.

The patient is a 33-year-old woman who reported to the University of Utah Sarcoma Service Clinic for evaluation of a mass just above her left knee. The mass was associated with local pain, but she was otherwise asymptomatic. Bone scan and physical examination disclosed no other lesions. Radiologic examination disclosed a bone lesion most consistent with osteosarcoma (Fig. 1). Following biopsy, an en bloc resection of the distal femur was performed.

### Histopathology

Both the biopsy and resection specimen were fixed in 10% neutral buffered formalin. Five micron H&E-stained sections were prepared, and immunohistochemistry for cytokeratins AE1,3, CAM 5.2, vimentin, S-100 protein, CK 5/6, CK 7, CK 19, CK 20 and HER-2/neu was performed. The antibody AE1,3 was supplied by Boehringer Mannheim (Mannheim, Germany) and used at a dilution of 1:2800. CAM 5.2 was supplied by Novocastra (Newcastle on Tyne, UK) and used at a dilution of 1:40. Vimentin used at a dilution of 1:30, S-100 used at a dilution of 1:300 and prediluted Her-2/neu were all supplied by Dako (Glostrap, Denmark). CK 5/6 was supplied by Chemicon (Temecula, California) and used at a dilution of 1:400. CK 7 used at a dilution of 1:800 and CK 20 used at a dilution of 1:200 were supplied by Dako (Glostrap, Denmark). CK19 was supplied by Vision Biosystems (Norwell, Massachusetts) and was used at a dilution of 1:400.

**Figure 2.**
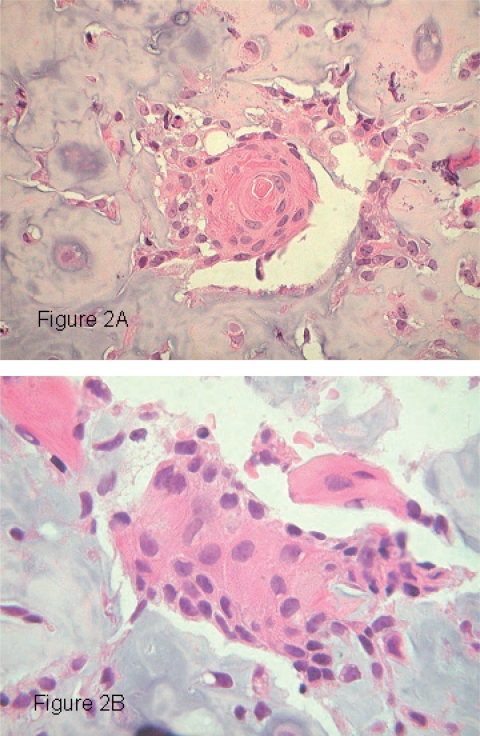
**A**) Keratin pearl composed of well differentiated neoplastic squamous cells within a zone of chondroid (H&E, ×40). **B**) Nest of malignant squamous cells showing intercellular bridges (H&E, ×100).

**Figure 3.**
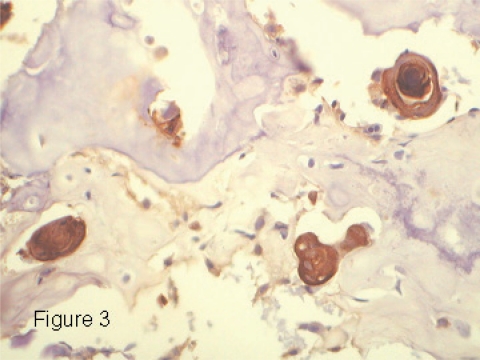
Immunohistochemical stain for cytokeratins AE1,3 demonstrating nests of positively staining cells.

Sections of the biopsy and resection specimen revealed an osteocartilaginous neoplasm in which were scattered small islands of epithelioid cells focally showing keratin pearl formation (Fig. 2A). These cells had enlarged hyperchromatic nuclei with a vesicular chromatin pattern and prominent nucleoli. The cytoplasm was eosinophilic. Intercellular bridges were visible (Fig. 2B). Immunohistochemistry for cytokeratins AE1,3, CK 7, CK 5/6 and CAM 5.2 revealed the nests of epithelioid cells to stain strongly for cytokeratins (Fig. 3). Immunohistochemical stains for vimentin, CK 19, CK 20, S-100 protein and HER-2/neu did not react with these cells. The majority of the neoplasm was composed of cartilaginous islands, woven bone and areas of direct tumor osteoid formation. A diagnosis of chondroblastic osteosarcoma was made.

## Discussion

Sarcomas showing epithelial differentiation with immunohistochemical demonstration of cytokeratin are well recognized and include synovial sarcoma and epithelioid sarcoma.^1^ Less commonly, other sarcomas may demonstrate minor populations of cytokeratin positive cells.^1^ Only synovial sarcomas and epithelioid sarcomas are believed to show true epithelial differentiation as characterized by the production of high molecular weight cytokeratins^1^, ^10^ and the production of clearly recognizable glandular or squamous elements.^1^ The epithelial differentiation and cytokeratin production seen in the other sarcoma types has been termed “anomalous”.^1^ This “anomalous” cytokeratin staining is characterized by a limited distribution in a subset of neoplastic cells and is confined to only a portion of the cytoplasm of those cells. Most sarcomas with “anomalous” cytokeratin production express cytokeratins limited to Moll’s catalogue 8 and 18 keratins corresponding to positive staining with CAM 5.2.^1^ Rare examples of osteosarcoma have been reported which show epithelial differentiation.^2^–^9^ Some reported cases have shown glandular or rosette-like features.^6^ The majority of cases have demonstrated immunohistochemical reactivity for epithelial membrane antigen and CAM 5.2.^6^, ^9^, ^11^ More rarely, osteosarcomas containing cells decorating with the antibody to cytokeratins AE1,3 have been reported.^8^ Most commonly, epithelial differentiation takes the form of small nests and clusters of polygonal epithelioid cells which lack definitive glandular or squamous differentiation. These osteosarcomas are referred to as epithelioid osteosarcomas^12^ and require distinction from metastatic disease.

Osteosarcomas with epithelial differentiation are relatively rare, and rarer still are those showing rosette-like, glandular or squamous differentiation. A small number of osteosarcomas have been reported with glandular or rosette-like differentiation and/or high molecular weight cytokeratin expression.^6^, ^8^, ^9^ Some of these osteosarcomas including our case have a chondroblastic morphology.^9^ Our case demonstrated clear-cut squamous differentiation including keratin pearl formation. This adds to the spectrum of epithelial differentiation reported in the literature and expands the potential differential diagnosis to include metastatic squamous cell carcinoma.

Primary bone neoplasms containing a low grade osteocartilagenous component and a cellular component showing epithelial differentiation raise the differential diagnosis of metastatic carcinoma with reactive bone formation, osteosarcoma with focal epithelial differentiation, and true carcinosarcoma. Carcinomatous metastases to bone are most commonly associated with a clinically or radiographically demonstrable primary lesion elsewhere in the body. The reactive bone associated with epithelial metastases usually shows less nuclear atypia than is characteristic of osteosarcomas showing focal epithelial differentiation. Many epithelial metastases present as osteolytic rather than osteoblastic lesions although metastases from some sites are characterized by osteoblastic metastases. Primary sites which may produce this radiographic picture include prostate, breast, stomach, colon, pancreas and lung, specifically pulmonary oat cell carcinoma.^13^ Careful clinical, radiographic and pathologic correlation is required to recognize the metastatic nature of the process. It should also be remembered that metastases are far more frequent than primary sarcomas in adults.

The presence of both mesenchymal and epithelial differentiation within a single neoplasm raises theoretical issues as to the origin of the bimorphic differentiation. When the epithelial portion of bimorphic differentiation is restricted to aberrant cytokeratin expression without cytoarchitectural signs of epithelial differentiation, two explanations have been profferred.^14^–^15^ Swanson suggested that the majority of aberrant cytokeratin expression within soft tissue sarcomas is due to technical artifacts resulting in false positive staining.^14^ This is an attractive proposal attributing the majority of aberrant cytokeratin expression in soft tissue sarcomas to excessive antigen retrieval, cross reactivity of antibodies or artifacts of fixation. However, the immunohistochemical finding of aberrant cytokeratin expression have been confirmed by other methods and cell biological evidence suggesting that cytokeratins are expressed in certain mesenchymal cells usually considered keratin negative.^11^ Cultured fetal fibroblasts have been induced to produce cytokeratins as shown by immunohistochemistry and western blotting techniques. Knapp and Franke^16^ have suggested that genes for cytokeratins 8 and 18 can escape expression regulation in certain circumstances and may be aberrantly expressed in some soft tissue sarcomas. The immunohistochemical findings of cytokeratins of the Moll’s classification 8 and 18 need not be a technical artifact but may represent true biological expression.

The osteosarcoma reported here and others described in the literature^6^–^8^ demonstrate epithelial differentiation extending beyond the expression of cytokeratins including ultrastructural evidence of desmosomes and monofilaments and glandular or rosette-like differentiation. Kramer et al.^8^ have reviewed several hypotheses formulated to explain the pathogenesis of these biphenotypic malignancies. Such tumors may arise from “cell rests” displaced from other tissues within the body, as collision tumors wherein a separate carcinoma and sarcoma amalgamate into an apparently single neoplasm or that carcinosarcomas represent extensive metaplasia of neoplasms fundamentally of epithelial origin.^8^ The most attractive hypothesis to explain biphenotypic tumors is that they arise from uncommitted multipotential stem cells. In this hypothesis, primitive mesenchymal cells differentiate in a nonrandom fashion resulting in a specific biphenotypic tumor.^8^ This hypothesis suggests that primitive mesenchymal cells can acquire epithelial morphology and express a variety of epithelial products including cytokeratins. Sarcomas with significant heterogeneity would appear most likely candidates for the focal production of the epithelial phenotype. The known heterogeneity of osteogenetic sarcomas would make them likely candidates for polyphenotypic differentiation.^2^,^6^

Our case illustrates the ability of osteosarcomas to show epithelial differentiation characterized both by cytokeratin expression and differentiation to squamous cells displaying keratinization and keratin pearl formation. Importantly, our case demonstrated higher molecular weight cytokeratin production (AE1, 3) than usually reported and squamous differentiation in place of a glandular phenotype. The present case raises the clinically important differential diagnosis between metastatic squamous cell carcinoma and osteosarcoma showing focal epithelial differentiation. Radiographic, clinical and morphologic features aided in the appropriate diagnosis of osteosarcoma in this case.
